# Relapsed Mantle Cell Lymphoma: Current Management, Recent Progress, and Future Directions

**DOI:** 10.3390/jcm10061207

**Published:** 2021-03-14

**Authors:** David A Bond, Peter Martin, Kami J Maddocks

**Affiliations:** 1 Division of Hematology, The Ohio State University, Columbus, OH 43210, USA; Kami.Maddocks@osumc.edu; 2 Division of Hematology and Oncology, Weill Cornell Medical College, New York, NY 11021, USA; pem9019@med.cornell.edu

**Keywords:** relapsed or refractory mantle cell lymphoma, novel agents, early progression of disease, cellular therapies

## Abstract

The increasing number of approved therapies for relapsed mantle cell lymphoma (MCL) provides patients effective treatment options, with increasing complexity in prioritization and sequencing of these therapies. Chemo-immunotherapy remains widely used as frontline MCL treatment with multiple targeted therapies available for relapsed disease. The Bruton’s tyrosine kinase inhibitors (BTKi) ibrutinib, acalabrutinib, and zanubrutinib achieve objective responses in the majority of patients as single agent therapy for relapsed MCL, but differ with regard to toxicity profile and dosing schedule. Lenalidomide and bortezomib are likewise approved for relapsed MCL and are active as monotherapy or in combination with other agents. Venetoclax has been used off-label for the treatment of relapsed and refractory MCL, however data are lacking regarding the efficacy of this approach particularly following BTKi treatment. Anti-CD19 chimeric antigen receptor T-cell (CAR-T) therapies have emerged as highly effective therapy for relapsed MCL, with the CAR-T treatment brexucabtagene autoleucel now approved for relapsed MCL. In this review the authors summarize evidence to date for currently approved MCL treatments for relapsed disease including sequencing of therapies, and discuss future directions including combination treatment strategies and new therapies under investigation.

## 1. Introduction

Mantle cell lymphoma (MCL) is a B-cell non-Hodgkin’s Lymphoma with characteristic deregulation of cell cycle arrest most commonly occurring due to gene translocation of *Cyclin D1* [1]. MCL is associated with a relapsing and remitting course with current treatment strategies, with generally shorter duration of remission with each subsequent line of therapy [2]. Frontline treatment for MCL, outside of investigational studies, is predominately with chemo-immunotherapy, with the incorporation of high dose cytarabine into induction shown to prolong duration of remission and the addition of rituximab maintenance shown to improve overall survival following autologous stem cell transplant [3,4,5,6]. Recent drug approvals have improved outcomes for patients with relapsed MCL, while leading to increasing complexity in terms of available treatment options and treatment sequencing. In this review, we will discuss the evidence supporting currently available therapies for relapsed/refractory (r/r) MCL, our approach to sequencing these therapies, and highlight future directions including novel agents under investigation for the treatment of r/r MCL. 

### 1.1. Prognostic Factors

Clinical and biologic factors provide prognostication for patients with MCL in the frontline and relapsed setting. The MCL International Prognostic Index (MIPI) is a risk score based upon clinical variables at diagnosis including age, performance status, lactate dehydrogenase, and white blood cell count that has been extensively validated [7,8,9,10] and provides prognostic information in the relapsed setting including among patients treated with BTK inhibitor (BTKi) [2,11,12]. Increased proliferation rate as measured by Ki67 immunostaining has been identified as a high-risk prognostic factor in both the frontline and relapsed setting [13,14,15,16,17]. The blastoid and pleomorphic histologic variants of MCL can occur at diagnosis or evolve at relapse in patients with prior classical histology (transformed MCL), and are associated with high risk disease [12,18], with patients with transformed MCL at highest risk for poor outcomes [19]. Molecular studies of MCL patient cohorts have identified a number of high-risk genetic features including mutations within *NOTCH1, NOTCH2, and KMT2D* and mutations or copy number loss of *CDKN2A* [20,21,22]. Furthermore, a robust body of literature demonstrates inferior outcomes for patients with *TP53* mutations, which are often associated with a high proliferative rate, following treatment either with intensive chemo-immunotherapy or BTKi [17,21,22,23,24,25]. 

In addition to these prognostic factors, emerging evidence supports duration of first remission as a robust prognostic marker in patients at first relapse. Among younger patients treated with intensive frontline therapy, duration of first remission less than 24 months was shown to be associated with inferior overall survival in both a retrospective cohort and validation cohort of patients treated in the MCL Younger study [26]. Consistent with these findings, recent work by our group has shown shorter duration of first remission to be associated with inferior survival after both intensive and less intensive frontline therapy in a large retrospective cohort [27]. While the clinical and biologic prognostic factors discussed in this section apply to both chemo-immunotherapy and BTKi treatment for patients with r/r MCL, these factors appear to be less predictive of response to chimeric antigen receptor T cell therapy (CAR-T) which has implication for the sequencing of treatment as discussed later in this review.

### 1.2. BTK Inhibitors

Three BTKi are FDA (United States Food and Drug Administration) approved for the treatment of r/r MCL, ibrutinib, acalabrutinib, and zanubrutinib. These orally administered drugs all share a common mechanism of action as irreversible covalent inhibitors of BTK, but differ in terms of kinase selectivity and pharmacokinetics and thereby toxicities and dosing schedule. The first in class BTKi ibrutinib is the least selective approved BTKi. Ibrutinib exhibits clinically relevant inhibition of kinases including ITK and EGFR in addition to BTK [28]. The efficacy of ibrutinib was demonstrated in the pivotal phase 2 PCYC-1104-CA trial enrolling 111 patients with r/r MCL treated with ibrutinib 560 mg daily, and confirmed in subsequent studies including the phase 2 MCL2001 study and the phase 3 MCL3001 study [29,30]. Pooled analysis of these three studies including 370 patients with r/r MCL treated with single agent ibrutinib has subsequently been published, providing robust characterization of the efficacy as well as toxicity profile of ibrutinib in this indication. The overall response rate (ORR) across the three pooled studies was 66%, including a complete response (CR) rate of 20%, with a median progression free survival (PFS) of 13 months [12]. Commonly observed toxicities included diarrhea in 40%, cough in 22%, nausea in 22%, and peripheral edema in 20%. Two serious toxicities observed at an increased frequency with ibrutinib include atrial fibrillation, which occurred in 5% of patients (Grade ≥ 3) in the pooled analysis and bruising or bleeding with a 5% incidence of major bleeding observed. The efficacy of single agent ibrutinib appears to be greater when used early in the sequence of treatment, with an ORR of 78% including 37% CR in patients receiving ibrutinib second line and a median PFS of 22 months versus 8 months among patients with two or more prior lines of treatment [25]. As previously referenced, mutations of the tumor suppressor *TP53* are associated with adverse outcomes with chemo-immunotherapy, and patients with *TP53* mutation treated with ibrutinib in prospective studies achieved a median PFS of only 4 months [25].

Acalabrutinib is the second BTKi to be granted accelerated approved in the United States for the treatment of MCL following a pivotal phase 2 study which enrolled 124 patients with r/r MCL (ACE-LY-004) [31]. Acalabrutinib is a highly selective BTKi with significantly less off target kinase inhibition relative to ibrutinib with an established dosing schedule of 100 mg twice daily [32]. In the phase 2 study in MCL, common toxicities included headache in 38% of patients, diarrhea in 31%, and myalgia in 21% of patients with no cases of Grade ≥3 atrial fibrillation, and major bleeding observed in 2% [31]. The ORR with single agent acalabrutinib was 80% (40% CR) with a median PFS of 20 months with extended follow-up [31,33]. Subgroup analysis demonstrated similar overall response rates but shorter duration of response (DOR) among patients with high-risk features including blastoid morphology (median DOR 14 vs. 26 months for classical morphology), high risk MIPI score (median DOR 10 vs. 29 months for low or intermediate MIPI), and Ki67% ≥50% (median DOR 15 vs. 26 months for ˂ 50%) [33].

Zanubrutinib, like acalabrutinib, exhibits greater selectivity for BTK relative to ibrutinib, and was the third BTKi granted accelerated approved for the treatment of r/r MCL [34]. Zanubrutinib monotherapy demonstrated single agent activity in the phase 1 BGB-3111-AU-003 study, with an ORR of 87% including CR in 30% among 37 patients with r/r MCL and a recommended phase 2 dose of 160 mg twice daily [35]. In a subsequent phase 2 study conducted in China, 86 patients with previously treated MCL were treated with zanubrutinib with an ORR of 84% including CR in 69% and a median PFS of 22 months [17]. While the BGB-3111-AU-003 study utilized CT for response assessment in the majority of patients, response assessment by PET was mandated in the subsequent phase 2 study; these differences in imaging modality likely explain the discrepancy in CR rate between the two zanubrutinib studies in relapsed MCL. This highlights an important consideration when comparing the CR rates reported in phase 2/3 studies of the approved BTKi, as the pivotal studies of ibrutinib utilized CT based response assessment, while the pivotal phase 2 studies of acalabrutinib and zanubrutinib mandated PET assessment. Commonly observed adverse events (any Grade) included neutropenia in 49%, thrombocytopenia in 33%, upper respiratory tract infection in 35%, and rash in 34% of patients. Major bleeding was observed in 3% of patients including one Grade 5 event (intracranial hemorrhage) with no cases of atrial fibrillation observed. Of 54 patients with baseline *TP53* sequencing performed, 15 patients had *TP53* mutated disease with an ORR of 80% and inferior PFS (15 versus 22 months) [17]. Direct comparison of the three currently approved BTKi in MCL is not currently available, however a head to head study of ibrutinib versus zanubrutinib for the treatment of Waldenström macroglobulinemia, demonstrated a lower incidence of bruising, diarrhea, muscle spasm, edema, atrial fibrillation, and pneumonia with zanubrutinib relative to ibrutinib with a higher incidence of Grade ≥3 neutropenia with zanubrutinib relative to ibrutinib [36].

### 1.3. Chemo-Immunotherapy

Bendamustine and rituximab (BR) is highly active in r/r MCL, with an ORR between 70–90% in prospective studies with a median PFS of approximately 18 months [37,38,39]. BR is widely used in the frontline setting, and it remains a reasonable option in patients with relapsed MCL without prior BR treatment. The combination of rituximab, bendamustine 70 mg/m^2^ day 1 and 2, and cytarabine 500 mg/m^2^ days 1–3 (R-BAC) has likewise been shown to be active in relapsed MCL [40], and has been shown to have a high response rate when sequenced after BTKi treatment with an ORR of 83% and median PFS of 10 months, including among patients with prior high dose cytarabine (64% of all patients) [41]. Dose reductions due to toxicity occurred in 56% of patients in this series, including 71% of patients treated with four or more cycles, highlighting the challenges of administering R-BAC at full dose intensity for six cycles in this setting. The combination of gemcitabine with rituximab and a platinum derivative, either cisplatin (R-GDP) or oxaliplatin (R-GemOx), is an alternative active chemo-immunotherapy option for r/r disease with ORR in greater than 80% of patients in phase 2 studies of both regimens [42,43]. Finally, the anthracycline containing regimen rituximab, cyclophosphamide, vincristine, doxorubicin, and prednisone (R-CHOP) is another active regimen in MCL [44,45], and while the expected duration of response to this this therapy is relatively short in the relapsed setting, in our experience this regimen can serve as a bridge to cellular therapy in select patients with refractory disease.

## 2. Lenalidomide and Bortezomib

The oral immunomodulatory drug lenalidomide is FDA approved for the treatment of relapsed and refractory MCL dosed 25 mg days 1–21 of 28 day cycle, with an ORR with single agent treatment between 28%-35% and median PFS ranging from 4–9 months in phase 2 and phase 3 studies [46,47,48,49]. Lenalidomide is associated with risk for hematologic toxicities including neutropenia and thrombocytopenia, gastrointestinal and dermatologic toxicities, and an increased risk for venous thrombosis. Lenalidomide has been studied in combination with rituximab in a phase 1/2 study with an ORR of 57% (36% CR) and a median PFS of 11 months [50]. The proteasome inhibitor bortezomib, dosed 1.3 mg IV or subcutaneously days 1, 4, 8, and 11 of a 21 day cycle, is FDA approved for the treatment of relapsed MCL based upon results from a pivotal phase 2 study with an ORR of 33% and median PFS of 6 months [51]. Bortezomib has also been studied in combination with rituximab and dexamethasone, with an ORR of 81% (44% CR) and a median PFS of 12 months [52]. Finally, the combination of rituximab, bortezomib, lenalidomide, and dexamethasone has been reported to be feasible and clinically active in a small series of heavily pretreated MCL patients sequenced after BTKi treatment [53], and in the authors’ experience has been a generally well tolerated and effective chemotherapy free regimen in this setting. However, it should be noted that the combination of lenalidomide and bortezomib has been studied in a prospective study which reported a high incidence of treatment discontinuation due to AEs and the toxicity profile is such that this combination would not be favored prior to BTKi treatment [54]. 

### 2.1. Venetoclax

Venetoclax is an orally administered selective BCL-2 inhibitor approved for the treatment of chronic lymphocytic leukemia and acute myeloid leukemia which demonstrated an ORR of 75% (21% CR) among 28 patients with MCL treated across all dose levels in a phase 1 study [55]. Given this high response rate to single agent therapy, venetoclax has been used off-label for the treatment of r/r MCL, although as the MCL patients treated in the phase 1 study had not received prior BTKi treatment, data regarding the efficacy of venetoclax sequenced following BTKi treatment are currently limited to retrospective reports. In a retrospective series of 20 patients treated in the United Kingdom with venetoclax after prior BTKi treatment through a compassionate use protocol, the ORR was 53% (18% CR) with a median PFS of 3.2 months [56]. In a separate retrospective series of 24 patients with r/r MCL, including 22 patients with prior BTKi treatment, an ORR of 50% (21% CR) was reported with venetoclax based therapy with a median PFS of 8 months [57]. Of note, the treatment given in this series was heterogeneous, with only half of patients receiving venetoclax as a single agent and others treated with venetoclax in combination with chemotherapy, BTKi, or obinutuzumab. Finally, a multi-center retrospective study was recently presented reporting outcomes for 81 patients with r/r MCL treated with venetoclax either as monotherapy in in combination with BTKi or monoclonal antibodies, with the majority (91%) of patients with prior BTKi treatment. The observed ORR in this series was 42% with a median PFS of only 3.7 months [58]. Notable toxicities of venetoclax include hematologic and gastrointestinal toxicity and infectious risk, and risk for tumor lysis syndrome has prompted recommendation for a ramp up dosing schedule beginning at a dosage of 20 mg daily, although ramp up schedules used in clinical practice with off-label venetoclax appear to be heterogeneous [55,59,60]. Our practice is to utilize weekly venetoclax ramp up dosing beginning at 20 mg daily when feasible [59]; however for patients with highly proliferative disease more rapid dose escalation with inpatient monitoring may be considered [61].

### 2.2. Cellular Therapies

Allogeneic hematopoietic cell transplant (allo HCT) has been shown to provide durable remissions in patients with r/r mantle cell lymphoma; however, allo HCT requires first achieving disease control and is associated with significant risks including risk for treatment related mortality and morbidity due to chronic graft versus host disease [62]. Despite these limitations, allo HCT remains a consideration for fit high risk patients with multiply relapsed disease, and appears to offer potentially durable benefit even among patients with very high risk biology including *TP53* mutated disease in one recent single center retrospective study [63]. 

More recently, chimeric antigen receptor T cell therapy (CAR-T) directed toward the B cell surface protein CD19 has emerged as a highly active treatment modality for the treatment of r/r MCL. Brexucabtagene autoleucel (formerly KTE-X19) is an autologous anti-CD19 CAR-T containing a CD28 signaling domain with a manufacturing process which includes removal of CD19 positive malignant cells during production. Brexucabtagene autoleucel was recently granted accelerated approved by the FDA for the treatment of adult patients with r/r MCL based upon results from the pivotal ZUMA-2 trial. In this phase 2, 74 patients with r/r MCL enrolled and underwent leukapheresis, with a total of 68 patients receiving brexucabtagene autoleucel following lymphodepleting chemotherapy [64]. Among the 68 patients receiving the drug, baseline characteristics included blastoid or pleomorphic histology in 31%, ≥3 prior lines of treatment in 81%, *TP53* mutation in 6 of 36 patients with available data, and prior BTKi treatment (acalabrutinib and/or ibrutinib) in all patients. The ORR among all 74 enrolled patients was 85% (59% CR) with an ORR of 93% (67% CR) among patients treated with brexucabtagene autoleucel with seven or more months of follow-up. Among these 60 treated patients, the 12 month PFS was 61% with median PFS not reached at time of study publication. Subgroup analysis demonstrated similar ORR and six month PFS among high risk subgroups including patients with *TP53* mutation, blastoid morphology, and high risk MIPI compared with patients without these high risk features. Observed toxicities among all treated patients included cytokine release syndrome (CRS) of any grade in 91% of patients which required administration of tocilizumab in 59% of patients and glucocorticoids in 22%. Grade 5 toxicities occurred in two patients (3%); one case of organizing pneumonia and one case of septicemia. Neurotoxicity of any grade occurred in 63% of patients, including Grade ≥ 3 toxicity in 31%. Persistent Grade ≥ 3 cytopenias were observed in 26% of patients beyond 90 days from treatment. While further follow-up is needed to determine the durability of response, brexucabtagene autoleucel represents a highly active treatment option for relapsed MCL, particularly among patients with progression following BTKi treatment and patients with high-risk features including blastoid morphology and *TP53* mutation. 

Lisocabtagene maraleucel (liso-cel; formerly JCAR017) is an autologous anti-CD19 CAR-T containing a 41BB signaling domain with a defined and separately administered CD4+ and CD8+ CAR-T dose currently under development for the treatment of B cell malignancies, including adult patients with r/r DLBCL and MCL. Results from the diffuse large B cell lymphoma patient cohort of the Phase 1/2 seamless design TRANSCEND NHL 001 study of liso-cel have recently been published [65], and preliminary results from 32 patients treated in the MCL cohort were recently presented in abstract form [66]. Baseline characteristics among the 32 treated MCL patients included blastoid morphology in 41%, *TP53* mutation in 22%, ≥3 prior lines of treatment in 69%, and prior BTKi treatment in 88%. Of note secondary CNS involvement by MCL was not exclusionary, with one patient with CNS involvement included among the 32 patients with preliminary results. Among the 32 treated patients, the ORR was 84% including 66% CR, with a median follow-up of 6 months and median PFS not reached. Grade 5 AEs occurred in two patients (6%); one case of TLS and one case of Cryptococcus meningoencephalitis. CRS of any grade occurred in 59% of patients, with 19% of patients requiring tocilizumab and 19% requiring glucocorticoid treatment for CRS or neurotoxicity. Neurotoxicity of any grade was reported in 34% including grade ≥3 in 13%. Grade ≥ 3 cytopenias persisting at day 29 were reported in 34% of patients. Complete and mature results from the TRANSCEND MCL cohort are awaited to better define the activity and determine durability of response; however, liso-cel appears to be a highly active treatment for relapsed MCL, with response reported in one patient with secondary CNS disease, and appears to be associated with lower incidence of CRS and neurotoxicity compared with brexucabtagene autoleucel. 

### 2.3. Sequencing of Treatment

While an expanding number of approved therapies are available for relapsed MCL, data regarding the optimal sequencing of these treatments for relapsed disease is limited. As previously discussed, prospective studies of the currently approved BTKi have consistently shown a longer PFS and duration of response when given earlier in the course of treatment. However, greater duration of response is also expected with chemo-immunotherapy with fewer prior lines of treatment, and treatments such as BR have not been compared head-to-head with BTKi in relapsed MCL. Recent analysis from a retrospective cohort of 261 younger patients with r/r MCL following frontline treatment containing high dose cytarabine demonstrated superior PFS for patients receiving either second line R-BAC or ibrutinib relative to BR or other second line treatment approaches [67]. Furthermore, while no difference was observed between ibrutinib or bendamustine based treatment among patients with duration of first remission of 24 months or more, treatment with ibrutinib was associated with superior PFS as well as OS relative to BR or R-BAC among patients with duration of first remission of less than 24 months. These data suggest that for patients with a short response or refractory to frontline intensive chemo-immunotherapy, BTKi or other targeted or cellular therapy approaches should be prioritized over alternative chemo-immunotherapy regimens. Prior reports described very poor outcomes for patients with progression on BTKi, with many of these patients heavily pretreated [68,69]. As previously discussed, a high response rate has been reported with R-BAC post BTKi with a median PFS of 10 months [41], and all patients treated in the pivotal phase 2 study of brexucabtagene autoleucel had been previously treated with BTKi and achieved an ORR greater than 90% and 12 month PFS of 61% [64]. One recent series reports poor outcomes in MCL patients with relapse following CAR-T treatment [70], and relapse after CAR-T treatment for MCL is a current unmet therapeutic need warranting further research. 

**Figure 1:**
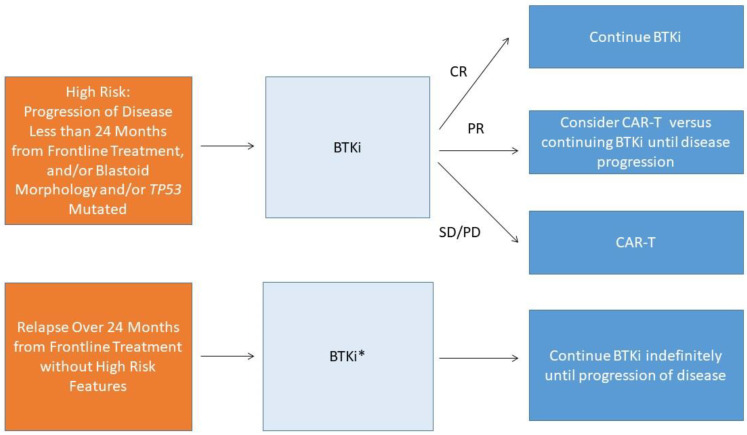
Authors’ Approach to Management of Mantle Cell Lymphoma at First Relapse. BTKi—acalabrutinib, ibrutinib, or zanubrutinib, CR- complete response, PR- partial response, SD- stable disease, PD- progressive disease, *- in select patients who favor time limited therapy and achieved prolonged duration of remission to frontline chemo-immunotherapy, would also consider chemo-immunotherapy as alternative option.

**Figure 2:**
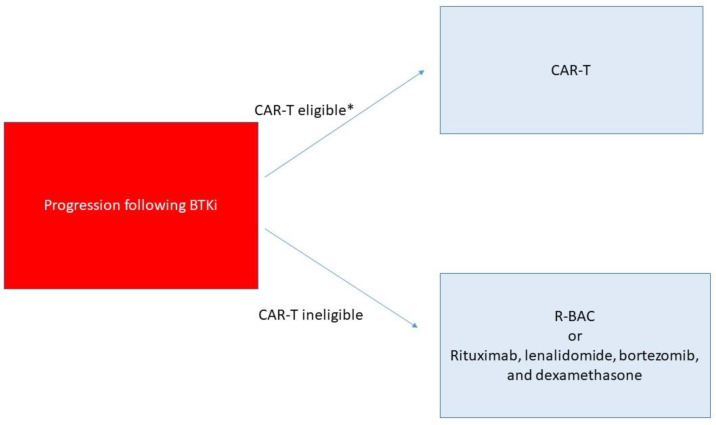
Author’s Approach to Management of Relapsed/Refractory Mantle Cell Lymphoma Following BTK inhibitor Treatment. BTKi—acalabrutinib, ibrutinib, or zanubrutinib, CAR-T- chimeric antigen receptor T cell therapy, brexucabtagene autoleucel is the only approved CAR-T treatment for mantle cell lymphoma at time of publication, R-BAC- rituximab, bendamustine, and cytarabine, *- patients with progression on BTKi require bridging therapy while awaiting CAR-T with either continuation of BTKi in cases with slow tempo of progression or with alternative therapies such as chemo-immunotherapy or venetoclax.

The authors’ general approach to second line treatment following frontline chemo-immunotherapy is depicted in Figure 1. While for patients with prior chemo-sensitive disease, particularly in patients who have not received prior bendamustine, BR or R-BAC are reasonable treatment options offering a time limited therapy, the authors generally prefer BTKi either in combination on a clinical study when available or as monotherapy as standard of care. This consideration is based upon both the expected duration of response to BTKi in the second line setting for patients with prior chemo-sensitive disease and the generally favorable side effect profile of BTKi relative to chemo-immunotherapy. For high-risk patients, including those with blastoid morphology, *TP53* mutated disease, and/or primary refractory disease, we prefer BTKi to chemo-immunotherapy second line, however the expected responses to BTKi are suboptimal in this setting and we recommend that these patients should also be evaluated for CAR-T. Whether to proceed with CAR-T while responding to BTKi versus reserving CAR-T until BTKi failure requires further prospective study and remains to be determined. Such patients should have close follow-up including attentive monitoring of blood counts and clinical symptoms for evidence of progression, and in patients with only stable disease at time of response assessment and high-risk features proceeding directly to CAR-T therapy should be strongly considered. For patients with progression of disease following BTKi treatment, the authors’ general approach to treatment is shown in Figure 2. Given the high response rates in this setting, for patients who are a candidate for cellular therapy approaches CAR-T is preferred in most instances. While CAR-T therapy use has been expanded in clinical practice to patients not meeting the strict eligibility requirements for the pivotal studies and there is not a strict upper age limit for these therapies [71,72], patients must have physiologic reserve to tolerate expected complications including CRS and thus patients with significant frailty or with severe end organ damage such as severe systolic heart failure are generally not candidates for this class of therapy. For patients progressing on BTKi, rapid disease progression has been observed in the author’s experience with BTKi discontinuation, and it is important to either continue BTKi or administer an alternative active treatment as bridging prior to leukapheresis and during CAR-T manufacturing. For patients who are ineligible for CAR-T and progress on BTKi, clinical trial participation should be prioritized when available. For standard of care treatment, R-BAC and rituximab, bortezomib, lenalidomide, and dexamethasone are both active options with choice of treatment dependent in part on patient characteristics and prior therapies received. 

## 3. Future Directions

### 3.1. Combinations

A wide range of combination treatment approaches have been previously studied for relapsed MCL, with selected results from previously published trials shown in Table 1 [16,73,74,75,76,77]. The combination of ibrutinib and rituximab was studied in a phase 2 trial enrolling 50 patients with r/r MCL and demonstrated an ORR of 88% (44%) with more favorable response rate noted in patients with Ki67 < 50% (ORR 100%, 54% CR) compared with high-risk patients with Ki67 ≥ 50% (ORR 50%, 17% CR) [16]. The triplet combination of ibrutinib, rituximab, and lenalidomide was studied in a phase 2 study enrolling 50 patients with r/r MCL [76]. This triplet combination was associated with a relatively high incidence of AEs including Grade ≥ 3 cutaneous toxicity (16%), infection (18%), and gastrointestinal toxicity (10%) and resulted in an ORR of 76% (56% CR). Among high-risk patients with *TP53* mutation, the response rates (73% ORR, 64% CR) were similar to *TP53* wild type patients, suggesting a potential role for this combination in this high risk patient subgroup. Finally, a phase 2 study of ibrutinib and venetoclax enrolled 24 patients with MCL (23 r/r) and reported an ORR of 71% with all responding patients achieving a best response of CR [75]. The overall and complete response rate among patients with Ki67 ≥ 30% was 56% (5/9 patients) and among *TP53* mutated patients was 50% (6/12 patients). Numerous phase 1 and 2 trials of combination approaches for r/r MCL are currently ongoing, shown in Table 2, and a large phase 3 study (SYMPATICO NCT03112174) comparing single agent ibrutinib to the combination of ibrutinib and venetoclax has completed accrual but mature data is not yet available. While combinations of targeted agents including BTKi have been shown to be feasible, and in some cases promising response rates have been seen in high-risk subgroups, to date no study has established the superiority of such approaches to single agent BTKi for relapsed MCL. 

### 3.2. Novel Agents

Non-covalent BTKi which target alternative sites within BTK distinct from the currently approved covalent BTKi are under development for MCL and other B cell malignancies [78]. The non-covalent BTKi LOXO-305 has been studied in the phase 1/2 BRUIN study which included 61 patients with r/r MCL, including 57 (93%) with prior treatment with covalent BTKi [79]. Among evaluable patients, the ORR was 52% including 25% CR, with similar response rates when limiting analysis to only those patients with prior BTKi treatment (also 52% ORR and 25% CR). The median DOR was not reached after a median of 6 months of follow-up among responding patients. While longer follow-up is needed to understand the long term side effect profile, the preliminary study results reported low rates of discontinuation due to AEs (1.5%) and infrequent Grade ≥ 3 AEs. A follow-up randomized phase 3 study (BRUIN-MCL-321, NCT04662255) of LOXO-305 versus investigator’s choice of ibrutinib, acalabrutinib, or zanubrutinib is planned to soon start enrollment to provide head-to-head comparison of the efficacy and toxicity profile of LOXO-305 compared with the currently approved covalent BTKi in patients with relapsed MCL.

Antibody drug conjugates (ADC) are another promising class of therapies for MCL and other B cell malignancies. While no ADCs are currently approved for MCL, preliminary results from a first in human phase 1 study demonstrate single agent activity in r/r MCL for the ROR1 targeted ADC VLS-101 [80]. Among 15 patients with previously treated MCL treated in a phase 1 study, VLS-101 had an ORR of 47% including 13% CR. Reported toxicities of VLS-101 included Grade 3 diarrhea in 9% and Grade ≥ 2 neuropathy in 35% of patients. Results from phase 2 studies are awaited to better define the activity of VLS-101 as well as other ADCs, which represents a promising emerging class of MCL therapy. 

Finally, while there are not currently any FDA approved PI3K inhibitors for the treatment of MCL, multiple drugs within this class have demonstrated activity in MCL [81,82,83] and this class of treatment remains of clinical interest for the treatment of relapsed disease. The selective PI3Kδ inhibitor parsaclisib was studied in the recently presented CITADEL-205 study in patients with r/r MCL, including cohorts with and without prior BTKi treatment. A total of 53 patients with relapsed MCL with prior ibrutinib treatment were treated across two dosing cohorts which varied in dosing schedule after week 8 of treatment [84]. The ORR among all patients was 25% including 2% CR with a median PFS of 3.7 months. Common AEs included diarrhea in 23% (any grade). Additionally, 108 patients with relapsed MCL without prior BTKi were treated across two dosing schedules [85]. Among BTKi naïve patients, the ORR was 70% (15% CR), with a median PFS of 11.1 months. With a longer median duration of exposure compared with the BTKi treated cohort, the rate of diarrhea (any grade) was 31% with discontinuation due to AEs occurring in 22% of patients. 

## 4. Conclusions

In summary, the number of approved classes of therapy continues to expand for relapsed MCL, with current treatment options ranging from chemo-immunotherapy to small molecule inhibitors to adoptive immunotherapy. Despite these advances, subsets of patients with aggressive disease biology or multiply relapsed disease continue to experience relatively poor outcomes with currently available therapies. With an expanding number of effective treatments, further study is needed to determine effective combination therapy approaches and to better inform sequencing of currently available therapies. Additionally, given that current MCL treatment approaches are generally not durable, additional active therapies are needed to continue to improve outcomes for patients living with this disease.

## Figures and Tables

**Table 1:** Summary of Published Combination Studies in Relapsed Mantle Cell Lymphoma.

Combination	Number of Patients	ORR% (CR%)	Median PFS	Toxicities
Rituximab and ibrutinib [16,73]	50	88 (44)	43 months	Gr 3/4 diarrhea 4%, Gr 3 atrial fibrillation 12%, Gr 3 Hypertension 2%
Obinutuzumab and ibrutinib [74]	9	78 (78)	2 year—89%	Gr 3 febrile neutropenia 11%
Obinutuzumab, ibrutinib, and venetoclax [74]	24	84 (67)	1 year—75%	Gr 3 diarrhea 12%, Gr 3 hypertension 4%
Venetoclax and ibrutinib [75]	24	71 (71)	1 year—75%	Gr 3 diarrhea 12%, Gr 3 atrial fibrillation 8%, Gr 3 respiratory infection 8%
Rituximab, lenalidomide, and ibrutinib [76]	50	76 (56)	16 months	Gr 3 Rash 14%, Gr ≥3 infection 24%, Gr 3/4 Diarrhea 12%
Ibrutinib and palbociclib [77]	27	67 (37)	2 year—59%	Gr 3/4 hypertension 15%, Gr 3/4 lung infection 11%, Gr 3/4 rash 7%.

**Legend:** ORR- overall response rate, CR- complete response rate, PFS- progression free survival, Gr- grade.

**Table 2:** Summary of Ongoing Combination Studies Enrolling Patients with Relapsed Mantle Cell Lymphoma.

Combination	Phase	Target Enrollment	Clinicaltrials.gov ID
Ibrutinib and venetoclax	Phase 3	362 patients	NCT03112174
Acalabrutinib and venetoclax	Phase 2	50 patients	NCT03946878
Venetoclax, lenalidomide, and rituximab	Phase 1/2	77 patients	NCT03505944
Carfilzomib, lenalidomide, and dexamethasone	Phase 2	59 patients	NCT03891355
Palbociclib and ibrutinib	Phase 2	61 patients	NCT03478514
Copanlisib and ibrutinib	Phase 1/2	45 patients	NCT03877055
Iaxazomib and ibrutinib	Phase 1/2	43 patients	NCT03323151
Iaxazomib and rituximab	Phase 2	24 patients	NCT04047797
Ibrutinib and tisagenlecleucel	Phase 2	20 patients	NCT04234061
Acalabrutinib and CD19 CAR-T cells	Phase 2	36 patients	NCT04484012
Loncastuximab and ibrutinib	Phase 2	161 patients *	NCT03684694
Polatuzumab vedotin, venetoclax, and rituximab	Phase 2	63 patients *	NCT04659044
Abexinostat and ibrutinib	Phase 1/2	40 patients *	NCT03939182
Lenalidomide, umbralisib, and ublituximab	Phase 1	42 patients *	NCT04635683
Venetoclax, obinutuzumab, magrolimab	Phase 1	76 patients *	NCT04599634
Lenalidomide and blinatumumab	Phase 1	44 patients *	NCT02568553
Pevonedistat and ibrutinib	Phase 1	30 patients *	NCT03479268

Legend: ID- identifier, * Includes other Non-Hodgkin’s lymphoma subtypes in addition to mantle cell lymphoma and/or alternative single agent or combination cohorts.

## Data Availability

Not applicable.
